# Exercise‐induced miR‐126 expression improves vascular health in prediabetes: A randomized controlled trial

**DOI:** 10.1113/EP093553

**Published:** 2026-03-19

**Authors:** Elif Yildirim Ayaz, Sadrettin Pençe, Sibel Kuraş, Emine Şeyma Denli Yalvaç, Banu Mesci, Özden Ezgi Üner, Fatoş Nimet Kaya, Berna Dincer, Ferruh Kemal Işman, Aytekin Oğuz

**Affiliations:** ^1^ Sultan 2. Abdülhamid Han Training and Research Hospital University of Health Sciences Internal Medicine Clinic Istanbul Turkey; ^2^ Istanbul Medeniyet University Department of Physiology Istanbul Turkey; ^3^ University of Health Sciences Department of Medical Biochemistry Istanbul Turkey; ^4^ Istanbul Medeniyet University Department of Cardiovascular Surgery Istanbul Turkey; ^5^ Prof. Dr. Süleyman Yalçın City Hospital Istanbul Medeniyet University Internal Medicine Clinic Istanbul Turkey; ^6^ Fatih Sultan Mehmet Training and Research Hospital Internal Medicine Clinic Istanbul Turkey; ^7^ Prof. Dr. Süleyman Yalçın City Hospital Internal Medicine Clinic Istanbul Turkey; ^8^ Istanbul Medeniyet University Department of Internal Medicine Nursing Istanbul Turkey; ^9^ Prof. Dr. Süleyman Yalçın City Hospital Istanbul Medeniyet University Department of Medical Biochemistry Istanbul Turkey

**Keywords:** atherosclerosis, carotid intima–media thickness, exercise, microRNA, miR‐126, prediabetes

## Abstract

In this study, we investigated whether a structured aerobic exercise programme could enhance microRNA‐126 (miR‐126) expression and improve subclinical atherosclerosis markers [carotid intima–media thickness (CIMT) and ankle–brachial index (ABI)] in individuals with prediabetes. In this 12 week, multicentre, assessor‐blinded, randomized controlled trial, 64 adults aged 18–60 years with newly diagnosed prediabetes were randomized into exercise and control groups. The exercise group performed supervised moderate‐intensity aerobic exercise programme three times per week (treadmill and cycling, 50%–70% of maximum heart rate), and both groups received standard lifestyle advice. Circulating miR‐126 expression was assessed via Δ*Ct* using qPCR at baseline and week 12. The mean age was 46.82 ± 7.94 years, and 75.0% were female. Within‐ and between‐group comparisons of Δ*Ct* miR‐126 were evaluated using a two‐way repeated‐measures ANOVA, demonstrating a significant main effect of time (*P* < 0.001) and a significant group × time interaction (*P* = 0.007). A significant group × time interaction was observed for both ABI and CIMT, with the exercise group showing an increase in ABI and a reduction in CIMT compared with the control group (ABI, *P* = 0.017; CIMT, *P* = 0.007). Correlation analysis revealed a positive association between change in Δ*Ct* and CIMT (r = 0.260, P = 0.045) and a negative correlation with ABI (r = −0.275, P = 0.034). A 12 week aerobic exercise intervention significantly increased miR‐126 expression and was associated with improvements in vascular markers of subclinical atherosclerosis, as evidenced by reduced CIMT and increased ABI. These vascular changes might be influenced, in part, by miR‐126‐related endothelial pathways among multiple mechanisms, highlighting the potential of miR‐126 as a biomarker and therapeutic target for early vascular protection in prediabetes.

## Abstract (highlights)

- **What is the central question of this study?**
  Is the exercise‐induced change in circulating miR‐126 associated with changes in subclinical atherosclerosis markers, including carotid intima–media thickness and ankle–brachial index, in individuals with newly diagnosed prediabetes?
- **What is the main finding and its importance?**
  A 12 week moderate‐intensity aerobic exercise intervention increased miR‐126 expression and was associated with reduced carotid intima–media thickness and improved ankle–brachial index. These findings suggest that exercise‐induced increases in miR‐126 might be associated with early vascular protective effects in prediabetes and that molecular adaptations might be reflected in improvements in vascular structure.

## 1 INTRODUCTION

Endothelial dysfunction is a crucial early event in the development of atherosclerosis and plays a central role in the pathogenesis of cardiovascular diseases (Clyne, 2021). It is characterized by reduced nitric oxide bioavailability, increased oxidative stress, vascular inflammation and impaired vasodilatation (Incalza et al., 2018; Zuccarelli et al., 2025). As a functional marker of vascular health, endothelial dysfunction often precedes structural changes in the arterial wall and serves as a precursor to clinically overt atherosclerosis.

Prediabetes, a condition of intermediate hyperglycaemia, is increasingly recognized as a metabolic state that promotes endothelial dysfunction, even before the onset of type 2 diabetes (Rooney et al., 2023). Individuals with prediabetes exhibit a pro‐inflammatory and pro‐atherogenic milieu, which accelerates vascular damage and increases the risk of cardiovascular events (Festa et al., 2005). With its rising global prevalence, prediabetes has become a major target for early cardiovascular risk reduction (Rooney et al., 2023).

Aerobic exercise is a well‐established intervention that improves endothelial function and reduces the progression of atherosclerosis (Gao et al., 2022). Through mechanisms involving enhanced nitric oxide synthesis, improved insulin sensitivity and attenuation of vascular inflammation, exercise exerts protective effects on the vasculature (Gao et al., 2022). However, the molecular mechanisms underlying these beneficial effects, particularly in the context of prediabetes, have not been elucidated fully.

Recent evidence highlights the role of epigenetic regulators, particularly microRNAs (miRNAs), in mediating the vascular benefits of exercise. MiRNAs are small, non‐coding RNAs that regulate gene expression post‐transcriptionally and have been implicated in various biological processes, including endothelial homeostasis, inflammation, lipid metabolism and glucose regulation (Asukai et al., 2017; Gao et al., 2022). Dysregulation of miRNAs is central to the pathogenesis of dysglycaemia and atherosclerotic cardiovascular diseases (Fasolo et al., 2019; Szabo & Csak, 2016; Vienberg et al., 2017).

MicroRNA‐126 (miR‐126) is one of the most abundantly expressed miRNAs in endothelial cells and plays a crucial role in maintaining vascular integrity, promoting angiogenesis and facilitating endothelial repair (Cao & Zhang, 2017). Studies have shown that circulating levels of miR‐126 are reduced in individuals with prediabetes and type 2 diabetes, and low levels are associated with increased cardiovascular risk (Dehghani et al., 2020; Y. Liu et al., 2014; Zampetaki et al., 2010). By enhancing endothelial cell proliferation and suppressing inflammatory signalling, miR‐126 might protect against atherogenesis.

Emerging research suggests that aerobic exercise can modulate circulating miRNA profiles, including upregulation of miR‐126 (Y. Ma, Liu et al., 2022). Previous interventional studies have demonstrated that combined lifestyle‐modification programmes involving exercise and diet can elevate serum miR‐126 levels in individuals with impaired fasting glucose and type 2 diabetes. However, few randomized controlled trials have isolated the independent effects of a structured aerobic exercise intervention on miR‐126 expression while simultaneously evaluating vascular outcomes, such as carotid intima–media thickness (CIMT) and ankle–brachial index (ABI). The aim of the present study, therefore, was to provide incremental insight by focusing exclusively on exercise‐induced miR‐126 modulation and its relationship to subclinical atherosclerosis markers in prediabetes.

The CIMT and ABI are non‐invasive, reliable and easily accessible markers of subclinical atherosclerosis. CIMT reflects cumulative exposure to atherosclerotic risk factors, whereas a reduced ABI is indicative of peripheral arterial disease and systemic vascular involvement (Aboyans et al., 2012; Can et al., 2014). Understanding whether exercise‐induced changes in miR‐126 are associated with improvements in these vascular indicators might provide new insight into the epigenetic mechanisms underlying exercise‐mediated vascular protection.

The aim of this study was to evaluate the effects of a structured aerobic exercise programme on miR‐126 expression, CIMT and ABI in individuals with prediabetes and to explore the interrelationship among these variables. We hypothesized that regular aerobic exercise increases miR‐126 expression and that this increase is associated with improvements in CIMT and ABI, reflecting changes in vascular structure and subclinical atherosclerotic burden. These vascular changes might be influenced, at least in part, by endothelium‐related mechanisms. Demonstrating such associations could advance our understanding of how exercise confers cardiovascular protection via epigenetic pathways in the prediabetic population.

## 2 MATERIALS AND METHODS

### 2.1 Study design

This study was designed as a 12 week, multicentre, assessor‐blinded, randomized controlled trial. The research was conducted between 1 July 2021 and 31 April 2023, at Sultan 2, Abdülhamid Han Training and Research Hospital, Fatih Sultan Mehmet Training and Research Hospital, and Prof. Dr. Süleyman Yalçın City Hospital. A total of 64 participants were included in the study. The study adhered to the ethical principles outlined in the International Council for Harmonisation of Technical Requirements for Pharmaceuticals for Human Use (Medicines Agency, 2021) and the *Declaration of Helsinki* (World Medical Association, 2013).

Ethical approval was obtained from the Clinical Research Ethics Committee of Istanbul Medeniyet University Göztepe Training and Research Hospital. The study was registered on the ClinicalTrials.gov Protocol Registration and Results System platform, with registration number NCT06809257. Before participation, all participants were informed about the purpose of the study, procedures, potential risks and benefits. Confidentiality principles were explained, and it was emphasized that participation was voluntary. Participants were informed that they could withdraw from the study at any time. Written informed consent forms, approved by the ethics committee, were obtained from all participants.

### 2.2 Participant selection

The study included individuals newly diagnosed with prediabetes according to oral glucose tolerance test and/or glycated haemoglobin (HbA1c) criteria (2 h plasma glucose levels of 140–199 mg/dL after a 75 g oral glucose tolerance test and/or HbA1c levels of 5.7%–6.4%), aged 18–60 years, physically inactive (International Physical Activity Questionnaire Level 1) and motivated to exercise. Participants were evaluated by a cardiology specialist (e.g., ECG and exercise ECG tests) to confirm their fitness for exercise and had no significant weight changes (±2.5 kg) within the last 6 months.

Exclusion criteria included severe medical conditions (e.g., advanced cancer, major neurological or endocrine disorders or respiratory failure), existing cardiovascular disease, body mass index of <19 kg/m^2^, metformin use within the last 6 months, use of antihypertensive, lipid‐lowering or other antidiabetic medications, hormone replacement therapy, life expectancy <1 year, human immunodeficiency virus positivity, substance abuse, or orthopaedic and cognitive disorders that could hinder exercise.

### 2.3 Randomization and blinding

**FIGURE 1:**
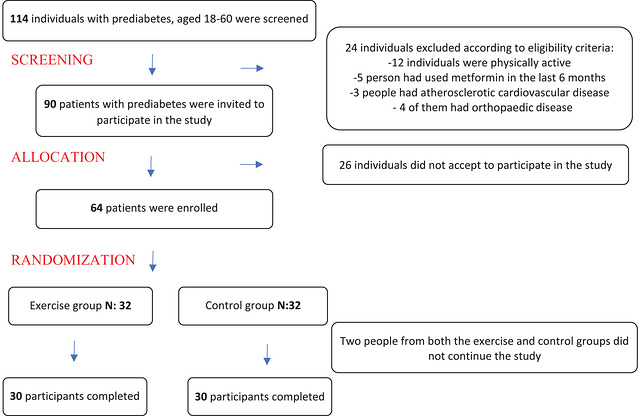
Flow diagram of participants.

The participant flow diagram is presented in Figure 1. A total of 64 participants who agreed to participate were randomized into intervention and control groups at a 1:1 ratio using computer‐generated block randomization (www.randomizer.org). Group allocation was not blinded to participants or implementers; however, serum analyses and statistical assessments were conducted in blinded conditions.

### 2.4 Follow‐up visits

Sociodemographic information on participants was recorded. Physical activity levels were assessed using the International Physical Activity Questionnaire (IPAQ), and dietary habits were evaluated with the Mediterranean Diet Adherence Scale (MEDAS). Anthropometric measurements were performed in accordance with standardized protocols.

### 2.5 Serum samples

Peripheral blood samples (6 mL) were collected from all participants at baseline and at the end of the 12th week after an 8 h overnight fast. Blood samples were delivered to the laboratory in yellow‐capped vacuum tubes within 10–20 min. Serum was separated by centrifugation at 800–1000 $g$ for 20 min at 4°C. Serum was separated within 40 min by centrifugation at 2000–3000 rpm for 20 min at +4°C. The separated serum was aliquoted into eppendorf tubes and stored at −80°C until analysis. Glucose, insulin, HbA1c, low‐density lipoprotein (LDL), high‐density lipoprotein (HDL), lipoprotein A and triglyceride levels were measured. The homeostatic model assessment of insulin resistance (HOMA‐IR) was calculated using the formula: glucose (mg/dL) × insulin (µU/mL) / 405.

### 2.6 Total RNA isolation

Total RNA isolation from serum samples was performed using a phenol–guanidine‐based lysis solution (QIAzol, Qiagen, catalogue no. 79306). This kit enables the isolation of RNA ≥17 nucleotides. The purity and concentration of isolated total RNA were measured.

### 2.7 Complementary DNA synthesis

Total RNA concentrations were normalized to 180 ng. Complementary DNA synthesis and subsequent analysis of miR‐126 expression were performed using the Single miRNA qPCR Assay kit (A.B.T. catalogue no. Q07‐01‐10) according to the manufacturer's protocol. MiR‐16‐5p served as the housekeeping gene. The cycle threshold (*Ct*) values for miR‐126 and miR‐16‐5p were used for analysis. The Δ*Ct* and ΔΔ*Ct* values were calculated as follows, and expression levels were analysed using the 2^−ΔΔ^ *^Ct^* method, as follows:

∆*Ct* = *Ct* (gene of interest) − *Ct* (housekeeping gene)

ΔΔ*Ct* = ∆*Ct* (treated sample) − ∆*Ct* (untreated sample)

Higher Δ*Ct* values indicate lower expression levels.

### 2.8 CIMT measurement

CIMT is a simple, inexpensive and valuable method for evaluating the cumulative effect of atherosclerotic risk factors. It serves as an important tool for the detection of subclinical atherosclerosis. CIMT measurements were performed by a cardiovascular surgeon using an M‐Turbo ultrasound system with a 5–12 MHz linear superficial probe. While the patient was lying in the supine position, the neck was turned ∼20° to the contralateral side. Measurements were taken from three different segments of the right and left carotid arteries: the common carotid artery, the carotid bifurcation and the first 2 cm of the internal carotid artery. Only the posterior wall was evaluated. Longitudinal measurements were obtained using B‐mode imaging by identifying the distance between the echogenic lines representing the lumen–intima interface and the media–adventitia interface. The mean CIMT was calculated as the average of three measurements taken from each side.

### 2.9 ABI

The ABI is defined as the ratio of the systolic blood pressure measured at the ankle to that measured at the brachial artery. It is a widely used, non‐invasive screening tool for the detection of peripheral arterial disease. Values between 0.9 and 1.5 are considered normal. An ABI value of <0.9 is associated with increased cardiovascular morbidity and mortality (Leng et al., 1996). In our study, the ABI was calculated by measuring systolic blood pressure at the ankle and brachial artery using a standard semi‐automatic sphygmomanometer and computing the ratio between these two values.

### 2.10 Exercise programme

Participants in the intervention group attended a structured aerobic exercise programme at the Diabetes and Obesity Sports Center for 12 weeks. The programme was supervised by medical doctors, sports trainers and physiotherapists and involved moderate‐intensity aerobic exercise (50%–70% of maximum heart rate) three times a week for 60 min per session. Maximum heart rates were calculated using the Karvonen formula (220 ­­ age), and pulse rates were monitored with fingertip oximeters to ensure appropriate intensity levels.

The exercise programme was structured in three phases: the first 4 weeks targeted 50% of the maximum heart rate, the 4th–8th weeks increased to 60%, and the 8th–12th weeks reached 70%. Participants exercised on treadmills for 30 min and on bicycle ergometers for another 30 min, with equipment settings adjusted by physiotherapists. Before and after each session, participants’ heart rate, blood pressure and oxygen saturation values were measured and recorded. Participants were monitored during the recovery period until their vital signs returned to baseline levels.

Breaks of 1 day were scheduled between exercise sessions, with a 2 day rest period after the third session. Attendance was monitored regularly, and participants were asked to report reasons for absences. In such cases, participants were instructed to walk outdoors or at a similar pace to treadmill activity for 1 h.

### 2.11 Lifestyle and dietary monitoring

Participants in both groups received general lifestyle recommendations, which included basic guidance on healthy eating and physical activity. The Mediterranean diet recommendation was not implemented as a structured or prescriptive dietary intervention; instead, it was provided as part of standard lifestyle advice. Baseline adherence to the Mediterranean diet was assessed using the MEDAS, and the same assessment was repeated at week 12 to monitor any changes throughout the intervention. Dietary adherence was therefore reassessed after the completion of the 12 week intervention and not during the weekly exercise sessions. In addition, habitual physical activity outside the supervised sessions was monitored using the IPAQ at baseline and at week 12. No individualized meal plans, dietary restrictions or targeted nutritional counselling were delivered, and participants were instructed to maintain their habitual diet throughout the study period. Therefore, dietary guidance was not considered part of the experimental manipulation, and any potential dietary or lifestyle variations were monitored but not intended to influence the primary outcomes.

### 2.12 Adherence monitoring and analytical approach

Exercise adherence was monitored through attendance logs and pre‐ and post‐session heart rate recordings. Adherence was defined as attending ≥85% of the scheduled sessions. Analyses were performed on participants who completed the intervention, following a per‐protocol analytical approach.

### 2.13 Control of potential confounders

Participants using antihypertensive, lipid‐lowering or antidiabetic medications were excluded at baseline to avoid pharmacological confounding. Baseline cardiovascular risk factors were comparable between the exercise and control groups. Therefore, no additional regression adjustment for these variables was performed. Potential variability in exercise intensity was minimized through supervised sessions and standardized training protocols.

### 2.14 Statistical analysis

Statistical analyses were performed using SPSS for Windows v.25.0 (IBM Corp., Armonk, NY, USA). Normality of continuous variables was assessed using the Shapiro–Wilk test. Continuous variables are presented as the mean ± SD, and categorical variables as numbers and percentages.

Baseline comparisons between the exercise and control groups were performed using Student's unpaired *t*‐tests for continuous variables and χ^2^ tests for categorical variables.

The primary outcome, circulating miR‐126 expression (Δ*Ct*), was analysed using a two‐way repeated‐measures ANOVA, with group (exercise vs. control) as the between‐subject factor and time (baseline vs. week 12) as the within‐subject factor. Main effects of group and time, in addition to the group × time interaction, were evaluated.

Changes in glucose control, lipid parameters and anthropometric measurements over time according to group were also analysed using two‐way repeated‐measures ANOVA.

For CIMT, baseline‐adjusted between‐group differences at week 12 were estimated using ANCOVA‐equivalent linear regression models, with follow‐up values as dependent variables and with baseline values of the respective outcomes together with group allocation included as fixed effects. Results are reported as unstandardized regression coefficients (*B*) with 95% confidence intervals (CI).

Associations between changes in miR‐126 expression and changes in CIMT were assessed using Spearman's correlation analysis, whereas associations with changes in ABI were evaluated using Pearson's correlation analysis.

All statistical tests were two‐sided, and a *P*‐value < 0.05 was considered statistically significant.

Based on power analysis conducted using existing literature, it was determined that including 30 participants in each group would provide 86% power, with a risk of 0.05. To account for potential data loss, the sample size was increased, and 32 participants were included in each group.

## 3 RESULTS

The mean age of the 60 participants included in the study was 46.82 ± 7.94 years, and 75.0% were female. Table 1 presents the distribution of sociodemographic characteristics, physical activity and dietary habits.

**TABLE 1:** Distribution of sociodemographic characteristics, physical activity and dietary habits.

		Control (*n* = 30)		Exercise (*n* = 30)		Test value	*P*‐value
Characteristic		*n*	%	*n*	%		
Sex	Male	8	26.7	7	23.3	0.089^†^	0.766
	Female	22	73.3	23	76.7		
Smoking status	No	22	73.3	21	70.0	0.082^†^	0.774
	Yes	8	26.7	9	30.0		
Age, years	Mean ± SD	47.37 ± 8.12		46.27 ± 7.86		0.533^‡^	0.596
IPAQ score, METs–min/week	Mean ± SD	726.21 ± 138.17		799.01 ± 226.80		−1.551^‡^	0.126
MEDAS score	Mean ± SD	5.37 ± 1.94		6.17 ± 1.97		−1.587^‡^	0.118

Abbreviations: IPAQ, International Physical Activity Questionnaire; MEDAS, Mediterranean Diet Adherence Screener.

^†^ χ^2^ test.

^‡^ Student's unpaired *t*‐test.

The mean attendance rate among participants in the exercise group was 88%. All participants attended >85% of the scheduled sessions and were therefore considered highly adherent. No adverse events occurred during the supervised exercise sessions.

### 3.1 Δ*Ct* miR‐126 values

**FIGURE 2:**
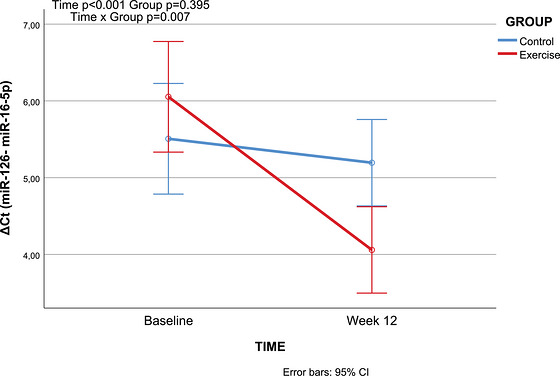
Interaction between time and group on Δ*Ct* miR‐126 levels.

Regarding miR‐126 expression, although no overall difference was found between groups, levels changed significantly over time across the entire cohort. Crucially, a significant group × time interaction revealed that the groups responded differently to the intervention. Specifically, the exercise group demonstrated a directional decrease in Δ*Ct* values from pre‐ to postintervention, indicating an upregulation of miR‐126 expression, a trend that was not observed in the control group (Table 2; Figure 2).

**TABLE 2:** Within‐ and between‐group comparisons of Δ*Ct* microRNA‐126.

	Exercise group (*n* = 30)		Control group (*n* = 30)				
Comparison	Pre‐exercise (baseline) Mean ± SD	Post‐exercise (12 weeks) Mean ± SD	Pre‐exercise (baseline) Mean ± SD	Post‐exercise (12 weeks) Mean ± SD	Group *F*/*P*/η^2^	Time *F*/*P*/η^2^	Group × time *F*/*P*/η^2^
ΔCt (miR‐126 vs. miR‐16‐5p)	6.05 ± 1.71	4.06 ± 1.33	5.51 ± 2.21	5.19 ± 1.72	*F* = 0.734 ***P* = 0.395** η^2^ = 0.012	*F* = 14.740 ***P *< 0.001** η^2^ = 0.203	*F* = 7.849 ***P* = 0.007** η^2^ = 0.119

*Note*: A two‐way repeated‐measures ANOVA was performed. Bold values indicate statistical significance (p < 0.05). Abbreviations: miR‐126, microRNA‐126; miR‐16‐5p, microRNA‐16‐5p.

### 3.2 Glucose control, lipid parameters and anthropometric measurements

Changes in glucose control, lipid parameters and anthropometric measurements over time according to group are shown in Table 3.

**TABLE 3:** Changes in glucose control, lipid parameters and anthropometric measurements over time according to group.

	Exercise group (*n* = 30)		Control group (*n* = 30)				
Parameter	Pre‐exercise (week 0) Mean ± SD	Post‐exercise (week‐12) Mean ± SD	Pre‐exercise (week 0) Mean ± SD	Post‐exercise (week 12) Mean ± SD	Group *F*/*P*/η^2^	Time *F*/*P*/η^2^	Group × time *F*/*P*/η^2^
Glucose, mg/dL	96.60 **± **8.06	90.66 ± 8.59	97.83 **± **10.60	94.29 **± **11.31	*F* = 1.218 *P* = 0.274 η^2^ = 0.021	*F* = 15.370 ***P *< 0.001** η^2^ = 0.209	*F* = 0.892 *P* = 0.326 η^2^ = 0.017
HOMA‐IR, mmol/L	2.81 **± **1.62	2.12 **± **1.22	3.03 **± **2.55	2.69 **± **2.01	*F* = 0.710 *P* = 0.403 η^2^ = 0.012	*F* = 9.309 ***P* = 0.003** η^2^ = 0.138	*F* = 1.090 *P* = 0.301 η^2^ = 0.018
HbA1c, %	5.96 **± **0.20	5.65 **± **0.31	5.94 ± 0.15	5.95 ± 0.32	*F* = 7.796 ***P* = 0.008** η^2^ = 0.1154	*F* = 14.894 ***P* ≤ 0.001** η^2^ = 0.204	*F* = 16.217 ***P *< 0.001** η^2^ = 0.219
Total cholesterol, mmol/L	5.28 ± 1.03	5.08 ± 0.93	5.24 ± 0.84	5.33 ± 0.92	*F* = 0.224 *P* = 0.638 η^2^ = 0.004	*F* = 0.476 *P* = 0.516 η^2^ = 0.007	*F* = 2.866 *P* = 0.096 η^2^ = 0.047
LDL cholesterol, mmol/L	3.19 ± 0.87	3.07 ± 0.76	3.12 ± 0.76	3.18 ± 0.76	*F* = 0.016 *P* = 0.900 η^2^ < 0.001	*F* = 0.117 *P* = 0.73 η*^2^* = 0.002	*F* = 1.395 *P* = 0.242 η*^2^* = 0.023
HDL cholesterol, mmol/L	1.41 ± 0.37	1.37 ± 0.50	1.47 ± 0.44	1.49 ± 0.46	*F* = 1.223 *P* = 0.273 η^2^ = 0.021	*F* = 0.116 *P* = 0.734 η^2^ = 0.002	*F* = 1.223 *P* = 0.273 η^2^ = 0.021
Triglyceride, mmol/L	1.50 ± 0.94	1.38 ± 0.68	1.51 ± 0.95	1.52 ± 0.94	*F* = 0.946 *P* = 0.733 η^2^ = 0.002	*F* = 0.834 *P* = 0.365 η^2^ = 0.014	*F* = 1.051 *P* = 0.310 η^2^ = 0.018
Lipoprotein (a), g/L	0.17 ± 0.18	0.19 ± 0.20	0.17 ± 0.17	0.17 ± 0.16	*F* = 1.410 *P* = 0.830 η^2^ = 0.001	*F* = 1.410 *P* = 0.240 η^2^ = 0.024	*F *= 0.287 *P* = 0.594 η^2^ = 0.005
Weight, kg	79.51 ± 13.61	76.25 ± 12.87	77.26 ± 15.18	76.04 ± 14.83	*F* = 0.114 *P* = 0.736 η^2^ = 0.002	*F* = 49.833 ***P *< 0.001** η^2^ = 0.462	*F* = 10.487 ***P* = 0.002** η^2^ = 0.153
BMI, kg/m^2^	29.89 ± 5.34	28.50 ± 5.25	30.02 ± 5.07	29.55 ± 5.05	*F* = 0.136 *P* = 0.714 η^2^ = 0.002	*F *= 50.547 ***P *< 0.001** η^2^ = 0.465	*F* = 9.872 ***P* = 0.003** η^2^ = 0.145
Waist circumference (cm)	98.81 ± 10.99	94.42 ± 10.15	98.13 ± 10.02	97.72 ± 9.24	*F* = 0.270 *P* = 0.605 η^2^ = 0.005	*F* = 12.038 ***P* = 0.001** η^2^ = 0.167	*F* = 8.233 ***P* = 0.006** η^2^ = 0.132

*Note*: A two‐way repeated‐measures ANOVA was performed. Bold values indicate statistical significance (p < 0.05).Abbreviations: BMI, body mass index; HbA1c, glycated haemoglobin, HDL, high‐density lipoprotein; HOMA‐IR, homeostatic model assessment for insulin resistance; LDL, low‐density lipoprotein.

Regarding glycaemic control, both groups showed a significant reduction in fasting glucose levels and insulin resistance (HOMA‐IR) over the 12 week study period. However, a distinct differentiation was observed in long‐term glucose regulation, in that the exercise group achieved a significant reduction in HbA1c levels, whereas no such improvement was noted in the control group, resulting in a significant group × time interaction.

In terms of lipid profiles, total cholesterol, LDL‐C, triglycerides and lipoprotein a remained relatively stable in both groups, without significant intergroup differences. Likewise, HDL‐C levels did not show significant changes throughout the intervention.

Significant improvements were observed in anthropometric markers primarily within the exercise group. Specifically, the exercise intervention led to a directional decrease in body weight, body mass index and waist circumference. The significant group × time interactions for these variables confirmed that the reduction in body mass and abdominal adiposity was associated specifically with the structured exercise programme rather than being a general temporal trend.

### 3.3 Changes in ABI and CIMT over time according to group

Changes in ABI and CIMT over time according to group are shown in Table 4.

**TABLE 4:** Changes in ankle–brachial index and carotid intima–media thickness over time according to group.

	Exercise group (n = 30)		Control group (*n* = 30)				
Index	Pre‐exercise (baseline) Mean ± SD	Post‐exercise (12 weeks) Mean ± SD	Pre‐exercise (baseline) Mean ± SD	Post‐exercise (12 weeks) Mean ± SD	Group *F*/*P*/η^2^	Time *F*/*P*/η^2^	Group × time *F*/*P*/η^2^
Ankle–brachial index	1.25 ± 0.09	1.30 ± 0.09	1.30 ± 0.13	1.28 ± 0.14	*F* = 0.265 *P* = 0.608 η^2^ = 0.005	*F* = 1.226 *P* = 0.273 η^2^ = 0.035	*F* = 6.043 ***P* = 0.017** η^2^ = 0.086
Carotid intima–media thickness, mm	0.641 ± 0.177	0.539 ± 0.106	0.614 ± 0.158	0.619 ± 0.143	*F* = 0.573 *P* = 0.452 η^2^ = 0.010	*F *= 6.278 ***P* = 0.015** η^2^ = 0.098	*F* = 7.682 ***P* = 0.007** η^2^ = 0.117

*Note*: A two‐way repeated‐measures ANOVA was performed. Bold values indicate statistical significance (p < 0.05).

The vascular markers of subclinical atherosclerosis responded differently to the intervention between the two groups. A significant group × time interaction was observed for the ABI, whereby the exercise group demonstrated a directional increase, whereas levels remained stable in the control group.

Likewise, CIMT showed a significant improvement following the intervention. The exercise group exhibited a notable reduction in CIMT, a trend that was absent in the control group. After adjusting for baseline values, the exercise group maintained significantly lower CIMT measurements at week 12 compared with the control group.

### 3.4 Relationship between difference of Δ*Ct* miR‐126 and difference of subclinical atherosclerosis indicators

Correlation analyses demonstrated a significant association between the epigenetic response and vascular outcomes. A positive correlation was observed between the change in Δ*Ct* miR‐126 levels and the change in CIMT (*r* = 0.260, *P* = 0.045), indicating that a reduction in Δ*Ct* (which represents an increase in miR‐126 expression) was associated with a decrease in carotid wall thickness. Conversely, the change in Δ*Ct* showed a significant negative correlation with the change in ABI (*r* = −0.275, *P* = 0.034), suggesting that the upregulation of miR‐126 was linked to improvements in theABI. These findings are summarized in Table 5.

**TABLE 5:** Relationship between difference of Δ*Ct* microRNA‐126 and difference of subclinical atherosclerosis indicators.

Parameter	Statistic	Difference of ankle–brachial index	Difference of carotid intima–media thickness
Difference of Δ*Ct* microRNA‐126	*r*	−0.275	0.260
	*P*	0.034^†^	0.045^‡^

^†^ Spearman's correlation analysis.

^‡^ Pearson's correlation analysis.

### 3.5 Baseline‐adjusted between‐group differences in CIMT at week 12

To evaluate the impact of the intervention further, between‐group differences in CIMT at week 12 were analysed using baseline‐adjusted models. After adjusting for baseline thickness, the exercise group demonstrated significantly lower CIMT values compared with the control group (*B* = −0.046; 95% CI: −0.077 to −0.015; *P* = 0.004; Table 6).

**TABLE 6:** Baseline‐adjusted between‐group differences in carotid intima–media thickness at week 12.

	Unstandardized coefficients	95.0% Confidence interval for *B*		
Index	Adjusted between‐group difference (*B*)	Lower bound	Upper bound	*P*‐value
Carotid intima–media thickness, mm	−0.046	−0.077	−Bold values indicate statistical significance (*p* < 0.05).0.015	**0.004**

*Note*: Baseline‐adjusted between‐group differences were estimated using ANCOVA‐equivalent linear regression. Bold values indicate statistical significance (p < 0.05).

### 3.6 Lifestyle and dietary adherence

Baseline and postintervention MEDAS scores and IPAQ physical activity levels outside the supervised sessions were comparable between groups. No significant differences were observed in changes in dietary adherence or unsupervised physical activity levels during the 12 week period (*P* = 0.277).

### 3.7 Quality control and assay variability

All qPCRs were performed in duplicate to ensure reproducibility. Intra‐ and interassay coefficients of variation for Δ*Ct* values were determined using randomly selected samples. The intra‐assay coefficient of variation was <2%, and the interassay coefficient of variation was <3%, indicating high measurement precision. The reference gene (miR‐16‐5p) showed stable expression across all samples (average expression stability value, M ≤ 0.5), confirming the reliability of normalization. The reference gene (miR‐16‐5p) showed stable expression across all samples (M value ≤ 0.5), confirming the reliability of normalization.

## 4 DISCUSSION

This study demonstrated that a structured aerobic exercise programme significantly increased miR‐126 expression in individuals with prediabetes and that this increase was significantly associated with reduced CIMT and increased ABI, both of which are indicators of subclinical atherosclerosis. Using a two‐way repeated‐measures ANOVA, we showed that changes over time differed significantly between groups, supporting a true intervention‐related effect rather than a simple temporal trend. Our study is the first randomized controlled trial to evaluate the effects of exercise on miR‐126 in individuals with prediabetes. Although miR‐126 is predominantly expressed in endothelial cells and plays a crucial role in vascular homeostasis, the observed changes in CIMT and ABI should be interpreted as reflecting changes in vascular structure and subclinical atherosclerotic burden. These vascular changes might be influenced, at least in part, by endothelium‐related mechanisms.

This finding is consistent with existing literature, supporting the protective effects of exercise on vascular pathologies. Studies investigating the effects of exercise on miR‐126 in humans are limited. In a study evaluating both prediabetic and diabetic individuals, lifestyle modifications involving physical activity and dietary changes in prediabetics, in addition to insulin and lifestyle modifications in diabetics, led to increased miR‐126 levels compared with baseline (Y. Liu et al., 2014). Although consistent with our findings, the randomized controlled design of our study and demonstration of differences both at the 12th week and between groups contribute significantly to the literature. Additionally, our study included only a selected group of individuals with prediabetes.

The observed associations between exercise‐induced increases in miR‐126 expression and improvements in vascular markers, such as CIMT and ABI, suggest that miR‐126 might contribute to the modulation of early atherosclerotic changes. This supports the concept that aerobic exercise might exert epigenetic regulatory effects on endothelial homeostasis and vascular remodelling. Although the increase in circulating miR‐126 was statistically significant, its biological magnitude should be interpreted with caution. The observed reduction in Δ*Ct* reflects a moderate fold change that might still be physiologically relevant, because previous studies indicate that even modest elevations in miR‐126 can promote endothelial repair, reduce vascular inflammation and improve nitric oxide‐mediated vasodilatation (Fernández‐Hernando & Suárez, 2018). Further studies are needed to determine whether this magnitude of change is sufficient to translate into measurable clinical outcomes.

Our findings align with emerging evidence positioning miR‐126 as a pivotal regulator of endothelial integrity, vascular repair and metabolic adaptation. Circulating miR‐126 progressively declines from healthy states to subclinical atherosclerosis, paralleling reductions in nitric oxide bioavailability and endothelial function (Yao et al., 2022). Both myocardial and circulating levels of miR‐126 are inversely related to haemodynamic overload, underscoring its relevance to vascular homeostasis (Gallo et al., 2024). Intervention‐driven metabolic improvements, such as weight loss and SGLT2 inhibition, have been associated with restoration of miR‐126 expression, highlighting its responsiveness to endothelium‐protective therapies (Mone et al., 2023; Veie et al., 2023). Reduced circulating miR‐126 characterizes type 2 diabetes and ischaemic cerebrovascular disease, further confirming its role as a biomarker of vascular vulnerability (Hu et al., 2023). Mechanistic evidence shows that endothelial miR‐126 maintains re‐endothelialization and prevents restenosis via selective regulation of p27‐dependent vascular smooth muscle proliferation (Santulli et al., 2014), and activation of the PI3K/Akt and MEK/ERK pathways supports its pro‐angiogenic and anti‐apoptotic effects (Kong et al., 2020). Exercise‐related activation of endothelial AMPK prevents oxidative stress and vascular dysfunction, acting through overlapping molecular routes with miR‐126 signalling (Kvandová et al., 2023). Moreover, aerobic training mitigates hyperglycaemia‐induced ischaemia–reperfusion injury by restoring nitric oxide bioavailability and reducing oxidative stress (Grandperrin et al., 2023).

Collectively, these converging findings suggest that exercise‐induced upregulation of miR‐126 in prediabetes represents an adaptive vascular response engaging AMPK‐ and PI3K/Akt‐dependent pathways to enhance endothelial repair, counteract oxidative stress and preserve vascular function. However, given that the present intervention lasted only 12 weeks, it remains unclear whether these molecular and vascular adaptations are sustained over time. Future longitudinal studies with extended follow‐up are warranted to determine the durability of these endothelial benefits and the long‐term cardiometabolic protection mediated by miR‐126.

Acute effects of exercise were investigated in a study by Da Silva et al. (2015), which found that circulating miR‐126 levels increased 30 min after exercise. miR‐126, an indicator of endothelial integrity, was found to increase after exercise testing and 4 h of cycling exercise (Uhlemann et al., 2014). Exercise‐induced regulation of miRNAs has been shown to mediate cardiac regeneration (Bo et al., 2021). In mouse models, high‐intensity interval exercise increased miR‐126 levels, leading to angiogenesis in cardiac tissue (Rad et al., 2020). These findings support the hypothesis that the regulation of miRNAs mediates cardiac regeneration processes.

MiR‐126 is located in intron 7 of *EGFL7* and is expressed exclusively in endothelial cells and endothelial progenitor cells.  It targets vascular endothelial growth factor (VEGF) to enhance endothelial progenitor cell proliferation, migration and tube‐like structure formation. Additionally, it regulates the translation of specific proteins to maintain endothelial functions. Literature demonstrates that obesity, diabetes and exercise influence miR‐126 expression (Olivieri et al., 2015).

In individuals with type 2 diabetes, low miR‐126 levels are considered an epigenetic marker of cardiovascular complications and an independent predictor of long‐term mortality (Pordzik et al., 2021). Low plasma and coronary sinus levels of miR‐126 have been associated with left ventricular function and cardiac repair potential in individuals with heart failure (De Rosa et al., 2018). Hyperglycaemia is known to reduce miR‐126 concentrations, contributing to diabetic macroangiopathy and microangiopathy (Li et al., 2016). By increasing both circulating and intracellular miR‐126 levels, aerobic exercise might counteract these pathological mechanisms and restore endothelial function.

Exercise‐derived exosomes have been identified as important biological mediators that facilitate intercellular communication in the improvement of cardiovascular diseases (Lai et al., 2023). These exosomes carry specific miRNAs, such as miR‐126, and regulate various signalling pathways, including mitogen‐activated protein kinase (MAPK) and nuclear factor kappa B (NF‐κB), which protect the cardiovascular system (Lai et al., 2023). Additionally, exercise‐induced exosome release has been reported to reduce myocardial apoptosis, while promoting angiogenesis, microvascular density and left ventricular ejection fraction (Wang et al., 2024). These effects play a crucial role in reducing myocardial fibrosis and ischaemic damage.

Exercise triggers protective mechanisms, such as improving endothelial function, promoting angiogenesis and reducing inflammation by increasing miR‐126 levels. Chronic aerobic exercise enhances circulating miR‐126 levels in diabetic individuals, activates PI3K/Akt/eNOS pathways and supports vascular regeneration through VEGF/SPRED1 targets (C. Ma et al., 2018). Although acute exercise and high‐intensity interval training protocols also increase VEGF levels, their effects on miR‐126 might vary depending on the exercise protocol. In our study, a guideline‐based aerobic exercise programme was applied to evaluate the chronic effects of exercise, achieving significant increases with moderate‐intensity exercise of 180 min per week.

Exercise limits the expression of pro‐inflammatory cytokines by reducing NF‐κB activity. MiR‐126 suppresses inflammation by targeting the HMGB1/RAGE pathway and reduces the production of reactive oxygen species (J. Liu et al., 2021). These mechanisms play a crucial role in preventing diabetic vascular complications. Considering that prediabetic individuals are thought to have the onset of microvascular complications, this finding is significant. Additionally, miR‐126 activates the PI3K/Akt pathway to improve insulin sensitivity and facilitates GLUT4 translocation (Dastah et al., 2021). In this context, miR‐126 might mediate improved glucose metabolism. Exercise supports all these processes, regulating glucose homeostasis and mitigating metabolic complications of diabetes.

The demonstration of increased miR‐126 expression in association with structural vascular improvements suggests that miR‐126 could serve not only as a biomarker of endothelial integrity but also as a therapeutic target in prediabetic and diabetic populations at risk of atherosclerotic cardiovascular disease. However, further experimental and clinical studies are needed to gain a better understanding of the mechanisms of miR‐126 associated with different exercise protocols.

The beneficial effects of exercise on metabolic parameters, such as glucose levels, HbA1c, HOMA‐IR, blood lipids and anthropometric measurements, in individuals with prediabetes are well documented (Sun et al., 2024; Zeng et al., 2024). Consistent with this, our study also demonstrated significant metabolic improvements in the exercise group compared with both baseline and the control group. However, the epigenetic mechanisms linking these changes to vascular outcomes remain insufficiently understood. The change in miR‐126 emerged as an independent determinant of vascular improvement. Although metabolic markers are known to influence vascular physiology, their structural effects might require longer‐term stabilization to become detectable. Taken together, these results support the notion that miR‐126 might serve as a more immediate and sensitive biomarker of endothelial adaptation to exercise in individuals with prediabetes.

Additionally, the anti‐inflammatory and oxidative stress‐regulating effects of miR‐126 emphasize its potential as a protective molecule against cardiometabolic diseases (J. Liu et al., 2021). Our findings provide important clues that these mechanisms might be activated by exercise in prediabetic individuals.

An important limitation of the present study is that direct and established measures of endothelial function, such as brachial artery flow‐mediated dilatation, were not assessed. Although CIMT and ABI provide valuable information on vascular structure and subclinical atherosclerosis, they do not quantify endothelial function directly. Therefore, the mechanistic link between exercise‐induced changes in miR‐126 and endothelial function should be interpreted with caution. The 12 week duration of the exercise programme restricts the ability to evaluate the long‐term effects of the intervention, particularly regarding the sustainability of vascular protection and the persistence of miR‐126‐related molecular adaptations. Although MEDAS scores were available at baseline and at week 12, dietary adherence was not included as a covariate in the statistical models. This decision was made because the study design did not incorporate a structured dietary intervention, and no significant differences in MEDAS score changes were observed between groups. Nevertheless, future studies with comprehensive dietary monitoring might benefit from incorporating dietary adherence as a covariate to further refine the interpretation of exercise‐related vascular and molecular adaptations.

Our study focused exclusively on moderate‐intensity aerobic exercise; therefore, it remains unknown whether high‐intensity exercise could yield stronger effects. The selection of participants from only three hospitals in Istanbul, Turkey, limits the generalizability of the findings. Moreover, blinding was not feasible for the exercise interventions. Excluding individuals with serious health conditions, such as cardiovascular diseases or heart failure, might have introduced a healthy selection bias, limiting the applicability of findings to relatively healthier prediabetic individuals. The higher proportion of women in the study population might limit the generalizability of the findings, because sex‐related differences in endothelial biology and miR‐126 regulation could influence exercise‐induced vascular responses. Variability in the motivation levels of participants might also have influenced the outcomes, because highly motivated individuals were more likely to adhere to the programme. Despite observing meaningful associations between miR‐126 and vascular indicators, our limited sample size constrained more robust statistical modelling. Another limitation is that only miR‐126 was assessed, although several other miRNAs (e.g., miR‐21, miR‐155, miR‐210 and miR‐221/222) are also known to regulate endothelial function and vascular homeostasis (Fernández‐Hernando & Suárez, 2018). Therefore, the observed vascular improvements might reflect broader exercise‐induced miRNA responses rather than effects specific to miR‐126. Future studies incorporating a more comprehensive miRNA panel are warranted to elucidate the molecular pathways underlying exercise‐mediated vascular protection.

## 5 CONCLUSION

In conclusion, this study demonstrates that exercise increases miR‐126 expression in individuals with prediabetes and that this increase is associated with improved vascular markers, including reduced CIMT and increased ABI. These findings suggest that the endothelial function‐enhancing effects of exercise might be mediated, at least in part, by miR‐126. Understanding the epigenetic mechanisms underlying prediabetes and developing exercise‐based therapeutic strategies targeting miR‐126 could offer new avenues for the prevention of atherosclerosis and its complications.

## AUTHOR CONTRIBUTIONS

All data were collected in Türkiye. Elif Yildirim Ayaz, Banu Mesci and Aytekin Oğuz conceptualized and designed the study. Elif Yildirim Ayaz, Özden Ezgi Üner, Fatoş Nimet Kaya and Emine Şeyma Denli Yalvaç recruited the participants, conducted the experiments and collected the data. Elif Yildirim Ayaz wrote the first draft of the manuscript. Elif Yildirim Ayaz and Berna Dincer performed the statistical analyses. Ferruh Kemal Işman, Sadrettin Pençe and Sibel Kuraş supervised the laboratory analyses. All authors approved the final version of the manuscript and agree to be accountable for all aspects of the work in ensuring that questions related to the accuracy or integrity of any part of the work are appropriately investigated and resolved. All persons designated as authors qualify for authorship, and all those who qualify for authorship are listed. Elif Yildirim Ayaz is the guarantor of this work and, as such, had full access to all the data in the study and takes responsibility for the integrity of the data and the accuracy of the data analysis.

## CONFLICT OF INTEREST

None declared.

## Data Availability

The datasets generated during and/or analysed in the present study are available from the corresponding author upon reasonable request.
